# Novel, Precise, Accurate Ion-Pairing Method to Determine the Related Substances of the Fondaparinux Sodium Drug Substance: Low-Molecular-Weight Heparin

**DOI:** 10.3797/scipharm.1505-20

**Published:** 2015-07-22

**Authors:** Amol A. Deshpande, P. Madhavan, Girish R. Deshpande, Ravi Kumar Chandel, Kaviraj M. Yarbagi, Alok R. Joshi, J. Moses Babu, R. Murali Krishna, I. M. Rao

**Affiliations:** 1 Analytical Research, Custom Pharmaceutical Services, Dr. Reddy’s Laboratories Ltd., Bollaram road, Miyapur, Hyderabad-500049 (AP), India; 2 Department of Physical, Nuclear and Chemical Oceanology, Andhra University, Visakhapatnam-530003, India

**Keywords:** Fondaparinux sodium, HPLC-ELSD, Validation, Purity, Related Substances, Ion pair

## Abstract

Fondaparinux sodium is a synthetic low-molecular-weight heparin (LMWH). This medication is an anticoagulant or a blood thinner, prescribed for the treatment of pulmonary embolism and prevention and treatment of deep vein thrombosis. Its determination in the presence of related impurities was studied and validated by a novel ion-pair HPLC method. The separation of the drug and its degradation products was achieved with the polymer-based PLRPs column (250 mm × 4.6 mm; 5 μm) in gradient elution mode. The mixture of 100 mM n-hexylamine and 100 mM acetic acid in water was used as buffer solution. Mobile phase A and mobile phase B were prepared by mixing the buffer and acetonitrile in the ratio of 90:10 (v/v) and 20:80 (v/v), respectively. Mobile phases were delivered in isocratic mode (2% B for 0–5 min) followed by gradient mode (2–85% B in 5–60 min). An Evaporative Light Scattering Detector (ELSD) was connected to the LC system to detect the responses of chromatographic separation. Further, the drug was subjected to stress studies for acidic, basic, oxidative, photolytic, and thermal degradations as per ICH guidelines and the drug was found to be labile in acid, base hydrolysis, and oxidation, while stable in neutral, thermal, and photolytic degradation conditions. The method provided linear responses over the concentration range of the LOQ to 0.30% for each impurity with respect to the analyte concentration of 12.5 mg/mL, and regression analysis showed a correlation coefficient value (r^2^) of more than 0.99 for all the impurities. The LOD and LOQ were found to be 1.4 µg/mL and 4.1 µg/mL, respectively, for fondaparinux. The developed ion-pair method was validated as per ICH guidelines with respect to accuracy, selectivity, precision, linearity, and robustness.

## Introduction

Fondaparinux sodium is a novel antithrombotic agent, the first of a new class of selective factor Xa inhibitors. It has favourable pharmacokinetics including 100% bioavailability, low variability, and a mean terminal half-life of 17 hours for young and 21 hours for healthy elderly volunteers, enabling once-daily administration [1]. It is indicated for the prevention of venous thromboembolic events (VTE), which may lead to pulmonary embolism in patients undergoing major orthopedic surgery of the lower limbs (MOSLL) such as hip fracture surgery (HFS), major knee surgery (MKS), or hip replacement surgery (HRS) [2]. Studies on the prevention of VTE after orthopedic surgery demonstrated significantly improved efficacy over the other low-molecular-weight heparin enoxaparin, with a >50% reduced risk of VTE and a similar safety profile [2, 3]. In contrast to animal-sourced competitors, unfractionated heparin (UFH), and low-molecular-weight heparin (LMWH), fondaparinux sodium is manufactured completely by chemical synthesis. It is a chemically synthesized methoxy derivative of a natural pentasaccharide sequence, which is the active site of heparin that mediates the interaction with antithrombin [4]. It has a challenging pattern of O- and N-sulfates, specific glycosidic stereochemistry, and repeating units of glucosamine and uronic acids [5]. Fondaparinux sodium is a linear octasulfated pentasaccharide (oligosaccharide with five monomer units), derived from a complex chemical synthesis comprising more than 50 chemical steps and several purification steps. This molecule has five sulfate esters on oxygen (O-sulfated moieties) and three sulfates on a nitrogen (N-sulfated moieties). In addition, it contains five hydroxyl groups on a molecule which are not sulfated and two sodium carboxylates. Out of five saccharides, there are three glucosamine derivatives and one glucuronic acid and one L-iduronic acid [6]. Five saccharides are connected to each other in alternate α- and β-glycosylated linkages as shown in Fig. 1. Given the complexity of structure of fondaparinux sodium, many impurities can form in the course of synthesis. The purification process is capable of eliminating most of the impurities; however, not all of the impurities can be removed, but they can be controlled at an acceptable limit. Regulatory requirements for the identification, qualification, and control of impurities in drug substances and their formulated products are now being explicitly defined, particularly through the International Conference on Harmonization (ICH) [8, 9]. It is also recommended by ICH that all routine impurities at or above the 0.1% level should be identified and monitored through appropriate analytical methods [7–9].

**Fig. 1a:**
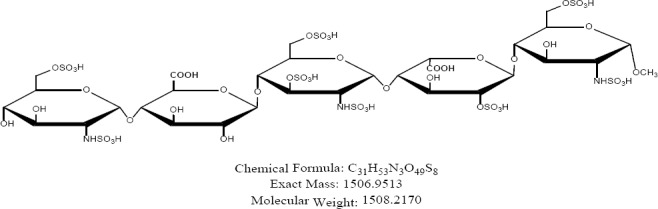
Fondaparinux

**Fig. 1b:**
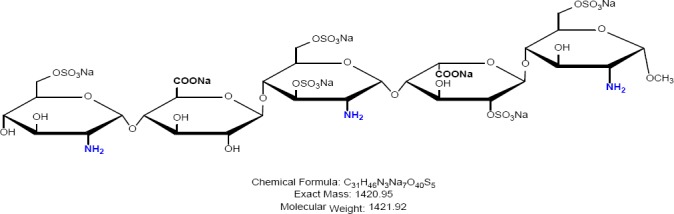
Impurity A

**Fig. 1c:**
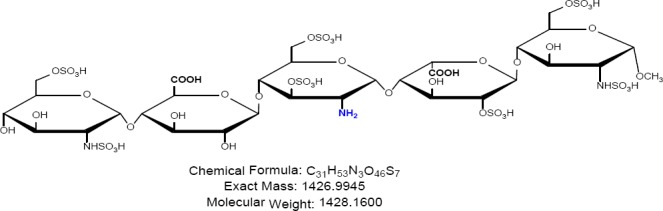
Impurity B

**Fig. 1d:**
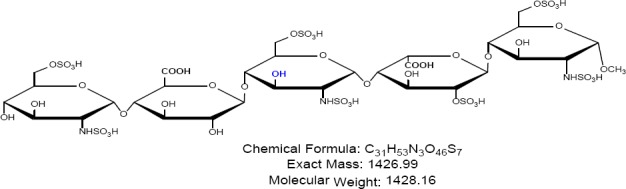
Impurity C

**Fig. 1e:**
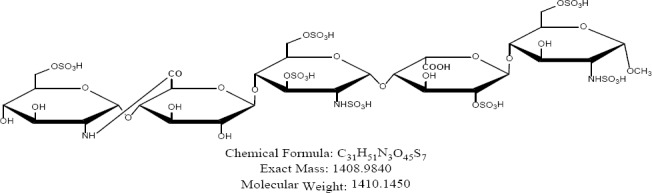
Impurity D

**Fig. 1f:**
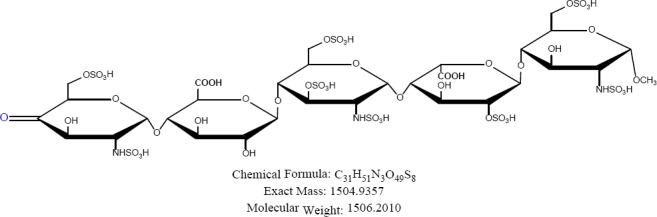
Impurity E

A literature survey revealed that only two analytical methods for the determination of purity of fondaparinux sodium have been reported. A strong anion exchange HPLC-based method is published in a US patent [10] without commenting on the stability-indicating nature of the method, in which the Carbopak PA1 (make: Dionex) column is used as stationary phase and 2 M sodium chloride was used as eluent. Another method is published [11] based on the radioactive detector (S35 labelling) and a low pressure HR 5/5 mono-Q (Make: Pharmacia) ion-exchange column. Fondaparinux was eluted in a gradient by varying the concentration of sodium chloride from 0.5 M to 2.0 M. Radioactive detectors are rarely available in analytical testing laboratories, making this method less viable. However, both of these studies lack comments on the limit of detection (LOD) and limit of quantitation (LOQ) of fondaparinux and its related impurities. This method also does not discuss the assay of the drug substance. Another strong ion-exchange HPLC-based analytical method recently appeared in USP Pharmacopeial Forum 39 (5). Due to the use of 2 M sodium chloride, the HPLC instrument requires frequent cleaning to avoid deposition of salts in the tubing, pump heads, or detector. Not using organic solvents also increases the chance of microbial growth which may lead to the clogging of the inlet frit and cause peak distortion for all peaks in the chromatogram. The proposed method overcomes these difficulties since the volatile buffer in combination with acetonitrile is used.

Many analytical methods are reported for the separation of heparin sulfate [12–19], which discuss the compositional analysis of heparin and low-molecular-weight heparin sulfate. An ion-pair chromatographic method [20] is reported for profiling the molecular entities of heparin materials, as well as for structural variability comparison of samples from various sources. However, no method so far is reported for related substances and the assay of fondaparinux sodium. Hence, a reproducible stability-indicating ion-pair HPLC method was developed for the quantitative determination of fondaparinux and its impurities, namely Impurities A, B, C, D, and E (Fig. 1B–F). This method was successfully validated according to the International Conference Harmonization (ICH) guidelines (Validation of Analytical Procedures: Test and Methodology Q2).

## Experimental

### Chemicals & Reagents

Samples of fondaparinux sodium were prepared in Process Research, Dr. Reddy’s Laboratories Ltd. HPLC grade acetonitrile (ACN) was purchased from Rankem (India), whereas diethylamine (DEA), triethylamine (TEA), and n-butyl amine (nBA) were purchased from Sigma Aldrich (India). The n-hexylamine (nHA), dibutyl amine (DBA), and acetic acid were purchased from Spectrochem pvt Ltd. (India); HPLC grade deionized water was prepared by a Milli-Q-Millipore Water System (Milford, MA, USA). The mobile phase solutions were filtered using 0.2-µm nylon membrane filters (Sartorius, France) on a solvent filtration apparatus.

### Instrumentation and Chromatographic Conditions: HPLC-ELSD

The HPLC system was an Agilent 1200 consisting of a quaternary gradient system, in-line degasser, autosampler, Variable Wavelength Detector (VWD), and evaporative light scattering detector or ELSD (Alltech, Model 3300). Data was processed using 1200 series Chemstation software (Agilent). Chromatographic separation was performed on the PLRPs analytical column (250 mm × 4.6 mm inner diameter, 5 µm particle size), (Varian PLRP-s,) maintained at 25ºC. ELSD was equilibrated at 100°C temperature with nitrogen gas flow of 2.0 L/min. The buffer was prepared as a mixture of 0.1 M n-hexylamine and 0.1 M acetic acid in deionized water. Mobile phase A was a mixture of buffer and ACN in the ratio of 90:10 (v: v) and mobile phase B was a mixture of buffer and ACN in the ratio of 20:80 (v: v). The mobile phases were run through isocratic mode with 2% mobile phase B between 0 min and 5 min, followed by a linear gradient as: 2%–85% of mobile phase B between 5–60 min, and finally 2% isocratic mobile phase B between 60 to 65 min) at a flow rate of 1.0 mL/min. The injector loop with 100 µL volume was used for injecting solutions.

### LC-MS Instrumentation

The ACQUITY UPLC system was connected to a standard ESI ionization source on a hybrid quadrupole time-of-flight mass spectrometer (Waters Q-TOF Premier Mass Spectrometer). The data acquisition was controlled by Mass Lynx 4.1 software (Waters). The spectra were acquired in negative ion mode. The following instrument settings were set for analyses in negative ion modes: source temperature 120°C, desolvation temperature 400°C, collision energy 5 eV. When operated in negative ion mode, the Q-TOF Premier Mass Spectrometer was set for the following instrument parameters: capillary voltage 2.8 kV, cone voltage 25 V, extraction cone voltage 4 V. Dibutylamine in combination with acetic acid was used as mobile phase for LC-MS experimentation.

### Preparation of Solutions

Fondaparinux sodium sample solution was prepared at the concentration of 12.5 mg/mL in HPLC grade deionized water. Stock solutions of individual impurity standards were prepared by dissolving the respective standard substance in deionized water to obtain a concentration of 1.0 mg/mL. The formulation of fondaparinux sodium was available in pre-filled syringes (PFS) in the strength of 2.5 mg/0.5 mL equivalent to a concentration of 5 mg/mL. Solution from ten PFSs was mixed thoroughly in a 5-mL volumetric flask and this solution was directly used for injection.

### Specificity

Specificity is the ability of the method to measure the analyte response in the presence of its potential impurities and degradation products [21]. Specificity of the method was demonstrated between process impurities *e.g*. the N-desulfated impurity of fondaparinux sodium (Impurity A), degradation products (Impurity B, C, D, and E) and related salts *e.g*. sodium chloride, sodium phosphate, and sodium sulfate, which are used either in the synthesis or in formulation of fondaparinux sodium. Stress studies were performed at the concentration 12.5 mg/mL of the drug substance to provide an indication of the stability-indicating property and specificity of the proposed method. Degradation was attempted under stressed conditions of photolytic degradation (UV light at 254 nm and visible light), heat (at 105°C), acid (0.1 N HCl at 27°C), base (0.5 N NaOH at 27°C), hydrolytic (60°C), and oxidation (10% H_2_O_2_ at 27°C) to evaluate the ability of the proposed method to separate fondaparinux sodium from its degradation products. The thermal study was performed for 7 days, the photolytic degradation study was for 11 days, whereas acid hydrolysis lasted 24 h; base hydrolysis was 2 h; hydrolytic degradation was 24 h, and oxidative degradation was 2 h. Peak homogeneity for fondaparinux sodium in the stressed samples was checked by scanning the peak through the mass detector, where no other mass number than fondaparinux sodium was observed in all stressed samples. The assay of stressed samples was performed by comparing peak areas with standard solution, thus the mass balance (% assay + % impurities + % degradation products) was calculated for the stressed samples.

### Impurity Isolation and Characterization

**Fig. 2:**
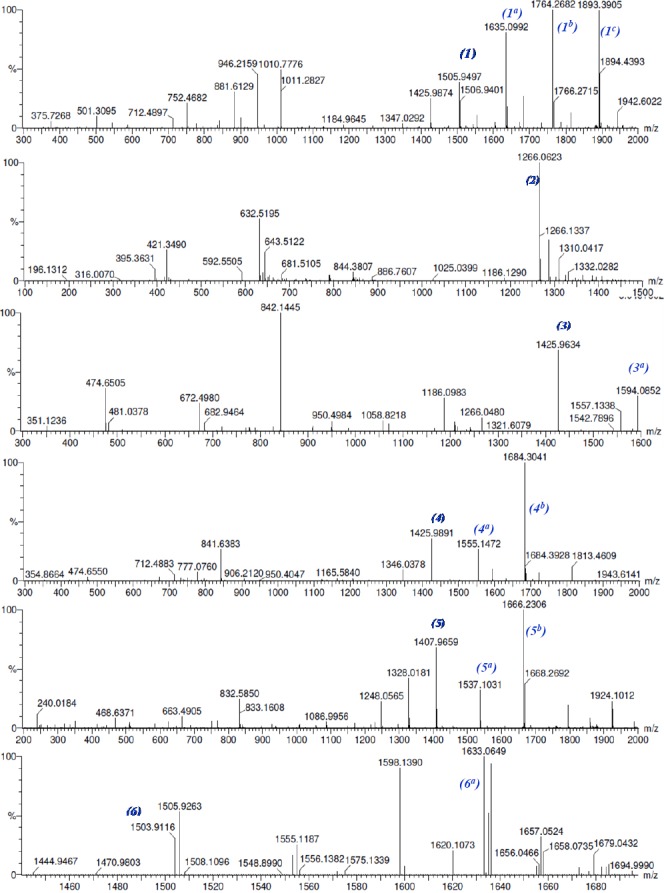
HRMS spectra of fondaparinux and its impurities: 1. Fondaparinux, 2. Imp A, 3. Imp B, 4. Imp C, 5. Imp D, 6. Imp E; a, b, and c represent mono, di, and tri DBA adducts of the impurity peaks, respectively.

The small quantities of impurities were isolated for identification, using PLRPs 250 × 4.6 mm analytical column and optimized gradient conditions. It was further optimized using PLRPs 250 × 21.2 mm semi-prep column to isolate the impurities for method validation. The degraded sample of each condition was run on the HPLC to identify the retention time of each degradation impurity. The impurities were then collected at the inlet of ELSD from the next run, as ELSD is a destructive technique. These collected impurities were passed through the strong anion-exchange (SAX) column to remove the buffer (hexylammonium acetate from mobile phase) followed by the gel-filtration column to remove the salts generated in SAX purification. All the major degradation impurities were isolated and used for the spiking study. The molecular mass and elemental composition of each impurity was determined using “time-of-flight (TOF)” mass analyzer. Mass spectral data displayed multiply charged ions; hence the spectral data was deconvulated (Fig. 2). Impurity structures were identified by heparinase cleavage, HR-MS, and NMR [45].

### Choice of Detector

Fondaparinux sodium is a low UV active molecule and detected at wavelength 210 nm by the UV detector [10]. Pre-column derivatization of a hydroxyl group is reported in literature [41], however, the presence of six primary and secondary hydroxyl groups add difficulty to achieve consistent derivatization. Carbohydrates are generally derivatized on the reducing end [42], but no free –OH group at the reducing end is present in fondaparinux, hence the option of derivatization was ruled out. Use of long chain amine inhibits the detection of fondaparinux at 210 nm as these have a higher UV cut-off. Fondaparinux and structurally related impurities are not separable in isocratic mobile phase conditions, hence the refractive index detector was also ruled out due to its non-compatibility to the gradient program, and ELSD was chosen for method development. The mobile phase chosen was a mixture of acetic acid and amine, which is volatile in nature and can be employed for ELSD.

### Reporting of Impurities

It is known that the relationship between the signal and the amount of mass present in ELSD is of a non-linear nature, hence the level of impurities cannot be reported by the area normalization method [44, 46–49]. To address this shortfall, low-level 3-point linearity was introduced (0.1% to 0.3% concentration of fondaparinux sodium with respect to nominal analyte concentration) and the concentrations of impurities in the test samples were interpolated from the regression parameters. The relationship between the area response and the analyte mass can be described by [46] & [50]:


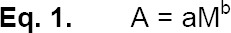


In the above equation, A is the area response of the detector, M the mass of the analyte present, a and b are values which depend on the analyte and chromatographic conditions. Since Eq. 1 can be transformed into:


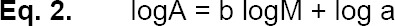


A log–log plot of the peak area versus analyte quantity will also yield a linear curve. After log–log transformation, linear regression can thus be used for calibration. Hence, the calibration curve was generated by plotting the log of the detector response vs. the log of the analyte concentration [46] and the content of impurities were calculated by using Equation 2.

### Optimization of ELSD Conditions

The nebulizing gas flow (N_2_) rate and drift tube temperature in an ELSD affect significantly the rate of response efficiency such as the detection sensitivity and the baseline stability [43]; hence the optimum conditions were investigated by observing the intensity of the fondaparinux signal. The drift tube temperature was checked from 60°C to 110°C, the drift tube temperature was fixed at 100°C where the satisfactory response was observed for fondaparinux and related substances. At 2.0 L/min gas flow rate, the optimum response was observed for the fondaparinux signal. The value of gain was kept at 1 as at the higher values, the baseline noise was significantly high.

### Mobile Phase Composition

The method for analysis of fondaparinux and its related substances was optimized using different mobile phase compositions. Acetonitrile was chosen as an organic modifier over methanol and tetrahydrofuran (THF) after initial trials because it improved the peak shape of fondaparinux sodium significantly and also produced better baseline values. Different gradients were tried to achieve the best separation between fondaparinux and related substances by using the buffer as mobile phase A and organic modifier as mobile phase B. The best separation was achieved with the gradient as the isocratic 12% B (between 0–5 min) followed by a linear gradient elution system as: 12–80% B (between 5–60 min), and finally 80% B isocratic (between 60–65 min) at a flow rate of 1 mL/min. Interestingly, as the percentage of acetonitrile was increased in gradient, relatively higher baseline noise was observed, which might be due to improper mixing of acetonitrile and buffer in the mixing chamber. To overcome this problem, the composition of mobile phase A and B was modified by mixing 10% organic modifier in mobile phase A and 20% buffer in mobile phase B. The gradient program was modified as: isocratic 2% B (between 0–5 min) followed by a linear gradient elution system as: 2–85% B (between 5–60 min), and finally 85% B isocratic (between 60–65 min).

### Method Validation

The proposed method has been validated for the assay and related substances by HPLC as per ICH guidelines [9].

#### Precision

Repeatability of the method was checked by analyzing six replicate solutions of 12.5 mg/mL fondaparinux sodium spiked with 0.15% of each of the known impurities and degradants. The percent RSD was calculated for each impurity for its content in terms of mg/mL. Intraday, interday, and analyst variations were studied to establish intermediate precision of the proposed method. Intraday precision was determined by repeating the repeatability experiment on the same day. The same procedure was followed for three different days to study interday variation (n = 18). The precision of the assay was evaluated by performing six independent assays of the test sample of fondaparinux sodium in comparison with a reference standard. The percent RSD of six assay results was calculated.

#### Sensitivity

The sensitivity of the method was proved by establishing the limit of detection (LOD) and limit of quantitation (LOQ) for fondaparinux sodium and its impurities and degradation products with a signal-to-noise ratio of 3:1 and 10:1, respectively. The LOD and LOQ were determined by injecting a series of diluted solutions having known concentrations of drug substance and impurities. The precision study was also carried out at the LOQ level by injecting six individual preparations of fondaparinux sodium and impurities at the LOQ concentration and by calculating the %RSD for the areas of each peak. Accuracy at the LOQ level was verified by injecting three individual preparations of fondaparinux sodium spiked with impurities at the LOQ level and by calculating % recoveries of each impurity.

#### Linearity and Range

To establish the linearity of the related substance method, solutions were prepared by diluting the impurity stock solution to obtain the required concentrations at seven different levels ranging from the LOQ to 200% (i.e. LOQ, 0.075, 0.12, 0.15, 0.18, 0.225, and 0.30%) with respect to the specification 0.15% w/w. The calibration curve was plotted taking the logarithmic values of concentration of the analyte on the X axis and logarithmic values of the respective area response on the Y axis. The correlation coefficient, slope, and y-intercept of the calibration curve were calculated.

#### Accuracy

For the determination of accuracy of the related substances method, a recovery study was carried out by analyzing the spiked samples. Known amounts of impurities were spiked in triplicate at three different concentration levels of 50%, 100%, and 150% with respect to nominal analyte concentration, and to a previously analyzed drug substance sample. The percentage of recoveries for each impurity was calculated.

#### Robustness

The robustness study was carried out to evaluate the influence of small, but deliberate variation in the chromatographic conditions. The factors chosen for this study which were potential variables in the operating procedures such as temperature of the column (±5 °C), strength of buffer (±0.01 M), flow rate (±0.1 mL/min), and gradient program were identified. Resolution between fondaparinux sodium and impurities was evaluated in the deliberately altered experimental conditions.

## Results and Discussion

### Method Development

As this drug substance is highly polar with labile sulfate groups and without chromophores, its method development has intrinsic challenges like the selection of stationary phase, selection of a suitable mobile phase, or ion-pair reagents, and the selection of an appropriate detector and optimization of its response.

### Column Selection

Retention is a process of the transfer of solute from a mobile phase environment into a stationary phase environment and hence, depends on the nature of both the mobile and stationary phases. The studies on variations in retention and selectivity among different reversed-phase columns shows [22] that differences in solute retention are correlated with three effects: (i) the effective phase ratio of the column as measured by the average retention of all solutes; (ii) the “polarity” of the bonded phase; and (iii) the dispersion solubility parameter of the bonded phase. Fondaparinux sodium, is a highly polar molecule in a polar stationary phase e.g. the HILIC column and carbohydrate columns were screened to get proper retention times of fondaparinux sodium. The literature suggests that retention can be enhanced by ion-pair formation with a non-polar stationary phase [23]; hence, conventional columns were tried as well.

The Benson column is a special carbohydrate column with 6% modification of Ca^++^ on the surface, and was tried with 100% aqueous and aqueous: ACN (95:5) composition. A higher composition of organic modifier will permanently damage the stationary phase; hence no more trials were performed on the Benson column. The Prevail column is made of hybrid stationary phase for carbohydrate analysis compatible with 100% organic modifier, which gives better retention of the polar molecule when used with a higher percentage of organic modifier. The trials were performed by using a water: acetonitrile composition in the ratio of 5:95, but it did not give satisfactory retention. Carbohydrate columns (refer to entry 2 and 4 on *Table 1*) did not retain fondaparinux sodium for sufficient time, which may be due to insufficient interactions of the stationary phase with negatively charged fondaparinux sodium. It was further proved when the HILIC column also failed to retain fondaparinux. For the conventional columns (entry no. 5, 6, and 7), ion-pair chromatography has become the obvious choice. After initial trials, the PLRPs column was chosen and better retention and resolution were achieved compared to the other two columns (Table 1).

**Tab. 1:**
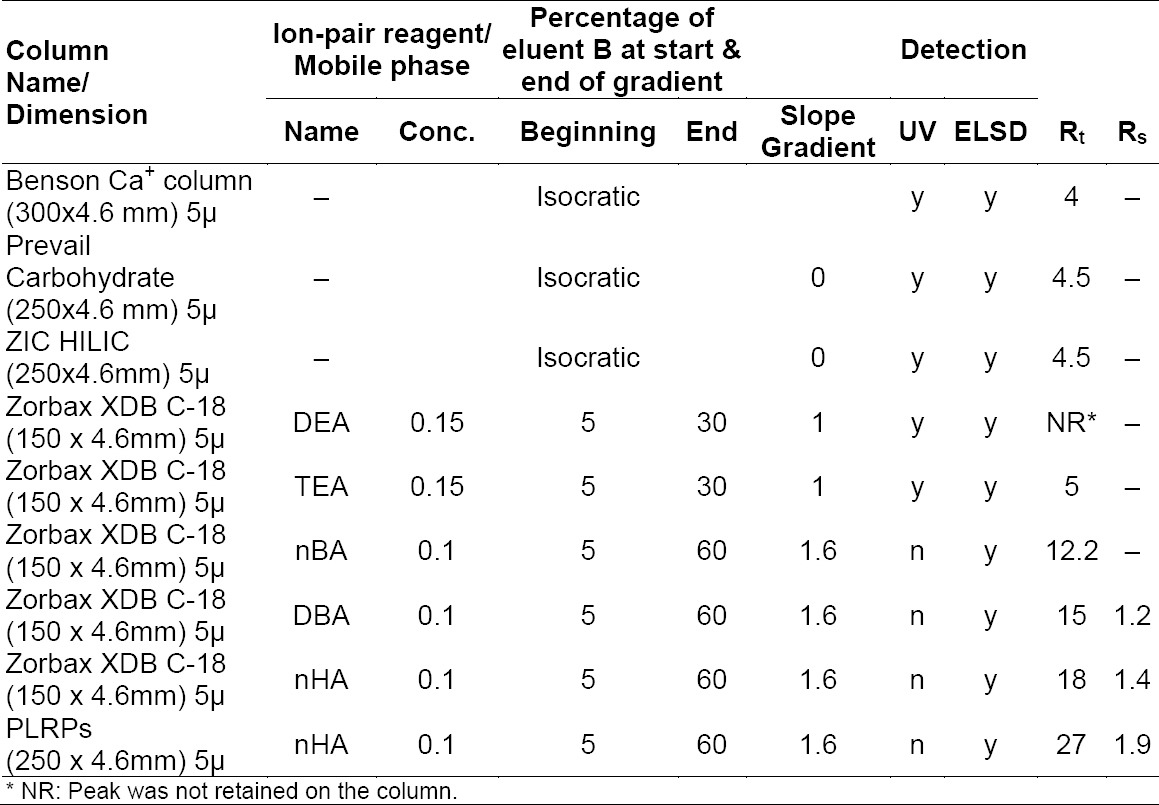
Gradient profiles used for each of the linear alkyl amines investigated as ion-pairing reagents for the separation of fondaparinux and Imp E.

### Selection of the Ion-Pair Reagent

Based on the few trials, it was evident that stationary phase alone will not help in retention. Moreover, fondaparinux sodium and its related substances are highly polar and negatively charged molecules, hence in the present work the number of small chain amines (DEA, TEA), long chain linear alkyl amines, tetra alkyl ammonium salts, and other buffers were evaluated (Table 1). The small chain amines were chosen due to the fact that they have lower UV absorbance at 210 nm, which enables use of the UV detection technique. Several theories are proposed to interpret the mechanism of ion-pair chromatography [24–34]. These can be divided into two categories: the ion-pair model and the dynamic ion-exchange model. The ion-pair model suggests that the analytes form ion-pairs with an amphiphilic ion of opposite charge in mobile phase, and the ion-pair complex is subsequently separated by following a reversed-phase mechanism. The dynamic ion-exchange model suggests that the stationary phase acts as a dynamically coated ion exchanger because of the adsorption of the amphiphilic ions, and analyte ions are retained and separated by the ion-exchange mechanism. Both of the theories can explain ion-pair chromatography successfully. Depending on the chromatographic conditions such as stationary phase, hydrophobicity of the ion-pair reagent, organic solvent type, and concentration, one mechanism might dominate over the other.

Linear aliphatic amines have been used as ion-pairing reagents for the separation of sulfonates [35]. They are readily available in high purity and are relatively more soluble in aqueous mobile phases compared to the corresponding tertiary amines with the same alkyl chains.

The choice of linear alkyl amine was influenced by the work of Doneanu C.E. *et al*. [20]. They screened a number of linear amines for separation of a different heparin and heparin disaccharide. Hexylamine was found as a suitable ion-pair in the combination of 1,1,1,3,3,3-hexafluoro isopropanol in their work. A.I. Gasco-López *et al*. [36] studied the effect of different amines as silanol masking agents on chromatographic behavior and they found hexylamine as the best silanol masking agent, provided a suitable concentration of amine is chosen. However, this is not the case with the present work as the PLRPs column was chosen for the method development work.

For the evaluations of different ion-pairing reagents, the mobile phases were composed of a linear alkyl amine as an ion-pairing reagent and acetic acid as a buffering acid. Since these linear alkyl amines do not carry a permanent positive charge, an acid was added to the mobile phase to protonate the amines. Its addition helped to lower the solution pH to convert all the free alkyl amines into ammonium ions. Protonation of amines was traditionally achieved using a volatile weak acid such as acetic acid [37–40]. The amines as an ion-pair reagent were used in excess in mobile phase (typically 30 mM-100 mM)

Retention of fondaparinux on special columns, e.g. the Benson, Prevail, and HILIC columns were close to 5 min and no resolution was achieved between fondaparinux and Imp E. Small chain amines, e.g. TEA and DEA did not improve the retention of fondaparinux. However, long chain amines proved to be a suitable option. The longer the chain length of the ion-pair reagent, the longer the time the compound takes to elute (Table 1) from the column. An increase in retention time was observed with increasing chain length, which attributes to an increase in the lipophilic properties of the ion-pair reagent as a higher degree of stationary phase coverage is achieved with increased chain length.

Dibutylamine and n-hexylamine in combination with acetic acid gave sufficient retention and resolution between the closely eluting impurity and fondaparinux sodium peaks, however the primary amine, n-hexyl amine, was chosen over di-butyl amine due to better miscibility with water.

The gradient conditions were further optimized to achieve the resolution between fondaparinux sodium and other process/degradation impurities.

### Specificity: Results from Forced Degradation Studies

Forced degradation samples under various conditions were analyzed at an initial concentration of 12.5 mg/mL of fondaparinux on HPLC using an ELS detector (Table 2). The retention time and relative retention time of impurities is presented in Table 3. The degradation products that were formed during the stress studies were confirmed by LC–MS. LC–MS analysis was performed as per experimental conditions given in Section 2.3. The stressed sample exhibited multiply charged ions (z>1) & dibutylamine adduct for all the impurities and fondaparinux, hence the data were deconvoluted.

**Tab. 2:**
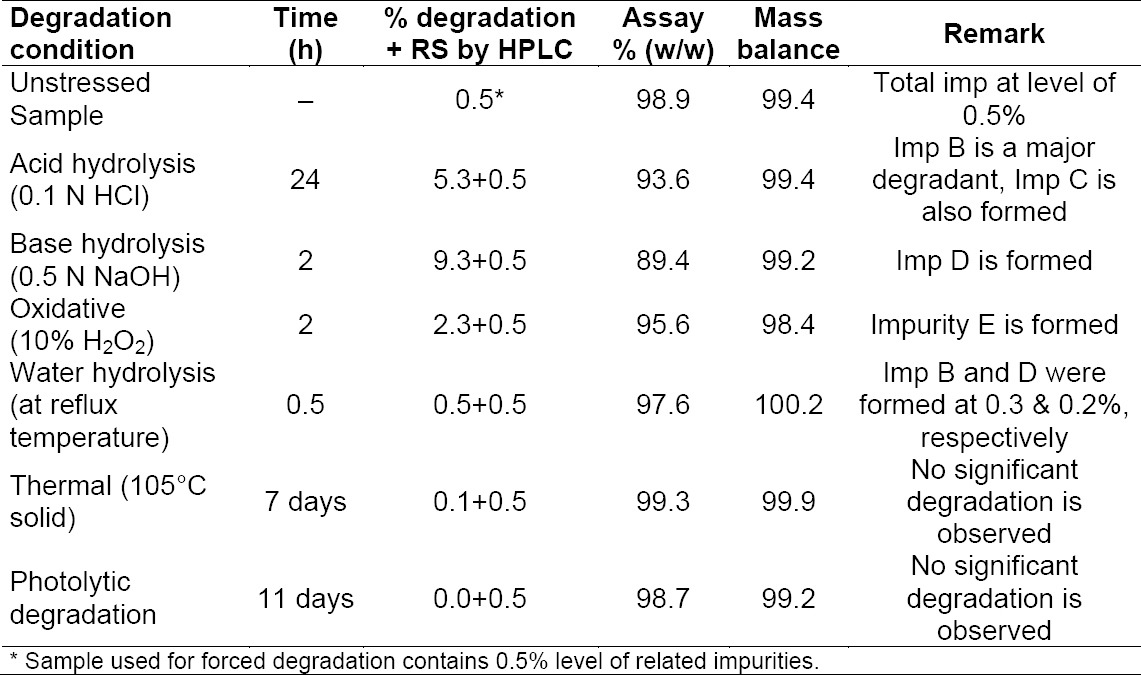
Summary of forced degradation results.

**Tab. 3:**
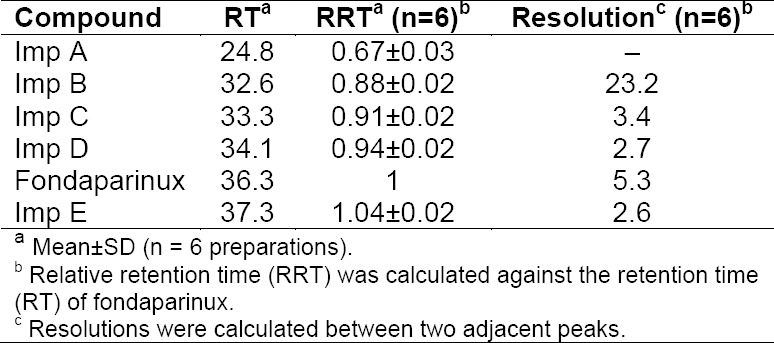
Resolution and retention time of impurities and fondaparinux.

Degradation was not observed when fondaparinux sodium was subjected to thermal degradation (105°C for 7 days) and photolytic degradation conditions. Significant degradation was observed when the drug substance was subjected to oxidation (10%

H_2_O_2_ for 2 h) leading to the formation of Imp E and acid hydrolysis (0.1 N HCl for approximately 24 h) leading to the formation of Imp B and Imp C. Imp B and Imp C are under-sulfated impurities which formed due to the loss of a -SO_3_ group. Impurity B formed due to the loss of one -SO_3_ from being attached to nitrogen of the C ring, and Impurity C was due to the loss of -SO_3_ from a primary hydroxyl of the C ring. Impurity B revealed a major degradant when compared to Impurity C. It was formed at the level of about 4% and Imp C at the level of 0.2%. Imp D formed due to desulfonation of the amine group on C2 of the E ring followed by lactonization of the D ring when the drug substance was subjected to base degradation (0.5 N NaOH for 2 h). Mass and elemental composition data reflect that there is loss of -SO_3_ and a water molecule.

The molecular mass of Imp E was determined to be 1503.9116 (as M-H) which is 2 atomic mass units less than fondaparinux. The difference of 2 atomic mass units could be due to the absence of two hydrogen atoms in fondaparinux.

The fragmentation experiments carried out for Imp E by LCMS/MS show a 2 atomic mass unit-loss from the parent ion, which indicates the oxidation of a secondary hydroxyl group to form a keto group, perhaps on the E ring. The difference of two atomic mass groups from the fondaparinux sodium molecule could not be determined by nuclear magnetic resonance spectroscopy. Hence, the structure of Imp E has been confirmed based only on mass spectroscopic data. Mass spectra of impurities is presented in Fig. 2.

**Fig. 3:**
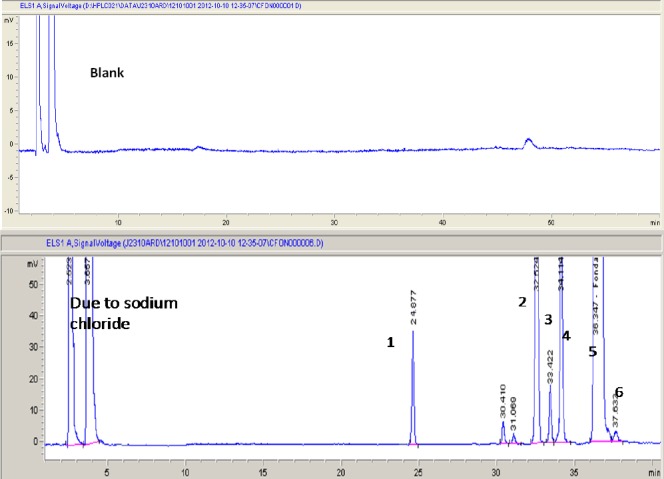
Blank chromatogram and chromatogram of specificity of fondaparinux in the presence of all the impurities: 1. Imp A, 2. Imp B, 3. Imp C, 4. Imp D, 5. Fondaparinux, 6. Imp E.

Assay studies were performed on the stressed samples against the fondaparinux standard. Mass balance results were calculated for all stressed samples and were found to be in the range of 98.4–100.2% which confirms the stability-indicating nature of the proposed method. A representative chromatogram of blank injection and specificity is presented in Fig. 3.

### Method Validation

An API containing Imp E was run before every method validation parameter and resolution between fondaparinux and Imp E and was recorded as system suitability. Also, a dilute solution of fondaparinux was run alongside.

#### Precision

The percent RSD for peak areas of the five impurities, namely Imp A, Imp B, Imp C, Imp D, and Imp E in the study of the repeatability was within 5.0. Results for intermediate precision are within 5.3%. These results demonstrate that the method is precise (Table 5).

**Tab. 4:**
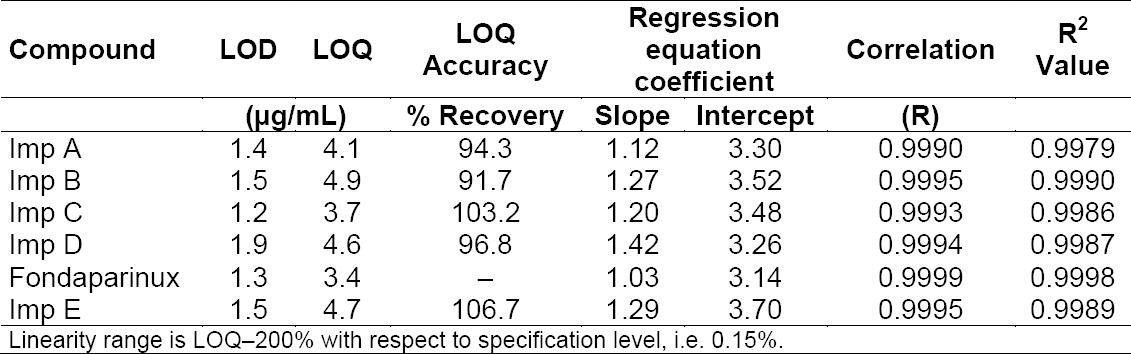
LOD, LOQ, and regression.

**Tab. 5:**
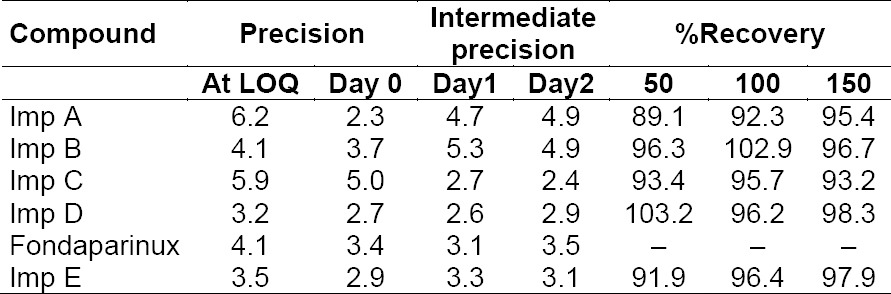
Precision and accuracy of fondaparinux and impurities.

#### Limit of Detection and Quantitation

Limit of detection, limit of quantitation values for fondaparinux, and its impurities are reported in Table 4. The percentage RSD for peak areas of fondaparinux and its related impurities at the limit of quantitation level are within 6.2%. The recoveries at the LOQ level are in the range of 91.7–107% (Table 4).

#### Accuracy/Recovery

Recovery of impurities from drug substances in the spiked studies ranged from 89.1 to 103.2% at three different levels as mentioned in Table 5.

#### Linearity

For all the impurities of fondaparinux, a linear calibration curve was obtained for the concentrations ranging from the LOQ to 0.30% and for fondaparinux from the LOQ to 200% (LOQ, 50, 75, 100, 150, and 200%) of the nominal sample concentration (12.5 mg/mL). The correlation coefficient obtained was >0.99 for all the components. The slope and Y-intercept values are also provided in Table 4, which confirmed good linearity between the log of peak areas and log of concentration.

#### Robustness

Under all the deliberately altered chromatographic conditions (flow rate, buffer strength, and column temperature), all peaks were adequately resolved and elution orders remained unchanged, which indicate that the method is robust. Resolution between fondaparinux and Impurity E was observed as system suitability in the parameter of the robustness study. It observed more than 1.7 in all of the robustness conditions. Results of this study are provided in Table 6.

**Tab. 6:**
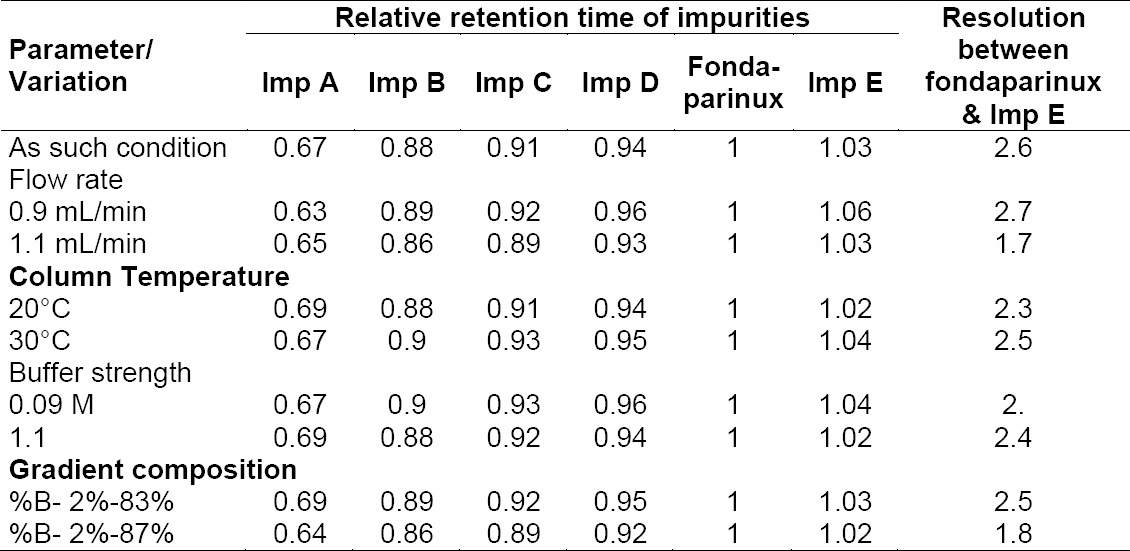
Results of robustness study.

## Conclusion

A novel stability-indicating liquid chromatography method has been developed using ELS detector. An attempt was made to explore the degradation behavior of fondaparinux by exposing it to ICH-defined stress degradation conditions. The developed method was able to separate Imp E (the closest peak to fondaparinux) with a minimal resolution of 1.7. The drug was found to be stable under water hydrolysis, thermal degradation, and photolysis conditions, whereas Imp B was a major degradant in acid hydrolysis, Imp D in basic hydrolysis, and Impurity E in oxidative degradation. The gradient RP-HPLC method developed and validated for the quantitative estimation of fondaparinux and related substances in active pharmaceutical ingredient was found to be precise, accurate, linear, robust, and specific. Thus, the developed method can also be used for the identification of stress degradation products along with routine quality control analysis. By reducing the concentration of n-hexylamine and acetic acid to 10 mM, it also can be used as mobile phase for mass experiments, which is an advantage of the developed method over the two other established methods.
